# Edge-enhanced infrared image super-resolution reconstruction model under transformer

**DOI:** 10.1038/s41598-024-66302-8

**Published:** 2024-07-06

**Authors:** Lei Hu, Long Hu, MingHui Chen

**Affiliations:** School of Computer and Information Engineering, Jiangxi Normal University, Nanchang, 330022 China

**Keywords:** Engineering, Optics and photonics

## Abstract

Infrared images have important applications in military, security and surveillance fields. However, limited by technical factors, the resolution of infrared images is generally low, which seriously limits the application and development of infrared images in various fields. To address the problem of difficult recovery of edge information and easy ringing effect in the super-resolution reconstruction process of infrared images, an edge-enhanced infrared image super-resolution reconstruction model TESR under transformer is proposed. The main structure of this model is transformer. First, in view of the problem of difficult recovery of edge information of infrared images, an edge detection auxiliary network is designed, which can obtain more accurate edge information from the input low-resolution images and enhance the edge details during image reconstruction; then, the CSWin Transformer is introduced to compute the self-attention of horizontal and vertical stripes in parallel, so as to increase the receptive field of the model and enable it to utilize features with higher semantic levels. The super-resolution reconstruction model proposed in this paper can extract more comprehensive image information, and at the same time, it can obtain more accurate edge information to enhance the texture details of super-resolution images, and achieve better reconstruction results.

## Introduction

Infrared imaging technology is widely used in the fields of security monitoring, automatic driving, and equipment thermal fault detection due to its features of good concealment, strong penetration, and high recognition. However, limitations associated with the infrared wavelength and imaging devices, the obtained infrared images generally have low resolution, making it difficult to meet the requirements of practical applications. Therefore, enhancing the resolution of infrared images holds significant research value and practical significance.

Image super-resolution (SR) reconstruction is a technique that involves restoring one or multiple low-resolution (LR) degraded images from the same scene into one or multiple high-resolution (HR) clear images. Compared to traditional SR reconstruction methods, deep learning-based approaches have become the mainstream due to their superior performance. These methods are primarily categorized into convolutional neural network (CNN)-based methods, generative adversarial network (GAN)-based methods, reference image-based methods, and transformer-based methods.

CNN possesses characteristics such as local connectivity and weight sharing, allowing CNN-based methods to better learn and represent image features. Dong et al.^1^ proposed SRCNN (image super-resolution using deep convolutional network, SRCNN) for the first time to achieve end-to-end image SR reconstruction using CNN. Shi et al.^2^ design ESPCNN (efficient sub-pixel convolutional neural network, ESPCNN), whose proposed sub-pixel convolution can efficiently solve the problem of upsampling enlargement during image reconstruction. Kim et al.^3^ devised the VDSR (very deep super-resolution) inspired by the residual learning concept, allowing the network to reach a depth of 20 layers. Lim et al.^4^ introduced EDSR (enhanced deep residual networks for single image super-resolution), which achieved a more concise image super-resolution reconstruction by removing redundant structures such as batch normalization layers^5^ from the residual modules. Zhang et al.^6^ introduced RCAN (residual channel attention network), which enhances the model's feature representation capability by introducing channel attention modules to emphasize important features. The improvement in the residual structure allows the model to stack more layers.

GAN-based methods utilize the adversarial nature of GAN structures to enhance the realism of image reconstruction Ledig et al.^7^ first introduced the concept of GAN into the SR reconstruction field with the proposed SRGAN (Super-Resolution Generative Adversarial Network). The images reconstructed by SRGAN exhibit richer texture information. Wang et al.^8^ proposed ESRGAN (Enhanced Super-Resolution Generative Adversarial Network), which utilizes residual dense blocks without batch normalization (BN) as the fundamental units. Additionally, improvements were made to the loss functions compared to SRGAN.

Transformer-based methods, with their distinctive self-attention mechanism, excel in effectively modelling distant dependencies among pixels and facilitating the interaction of contextual information within images. Liang et al.^9^ introduced SwinIR (image restoration using Swin Transformer), applying the Swin Transformer proposed by Liu et al.^10^ to the field of SR reconstruction. Conde et al.^11^ proposed Swin2SR (SwinV2 Transformer for Compressed Image Super-Resolution and Restoration), applying the Swin Transformer V2 introduced by Liu et al.^12^ to the field of SR reconstruction. The performance of Swin2SR is comparable to SwinIR in the classical field of SR reconstruction.

Infrared images commonly suffer from issues such as low resolution and poor contrast. During the SR reconstruction process, especially with high magnification factors, the difficulty arises from the limited information present in the input LR images. Edge restoration becomes challenging, leading to the potential occurrence of ringing artifacts. As a result, the performance of many existing SR reconstruction methods on infrared images is often less than satisfactory. To address this issue, we propose a transformer-based infrared image super-resolution reconstruction model with edge enhancement. The model comprises an SR backbone network and an edge extraction network. In the deep feature extraction of the SR backbone network, edge features are moderately introduced to enhance the sharpness of edges in the SR images.

The main contributions of this paper are as follows:

- An edge-enhanced infrared image super-resolution reconstruction model under transformer (TESR) is proposed. The deep feature extraction module is designed by introducing residual cross-shaped windows in the SR backbone network, and the edge features extracted by the edge-assisted network are additionally introduced in the deep feature extraction process to enhance the SR detail effect of infrared images with high magnification factors. This approach not only provides a larger receptive field but also leverages available edge information to learn and fill in missing pixels, reconstructing SR infrared images at different magnification factors.
- Aiming at the problems of insufficient extraction of detail features and lack of edge information in existing models, this paper designs a deep feature extraction module and an edge detection auxiliary network to jointly enhance texture and edge features. In the deep feature extraction module, the self-attention of horizontal and vertical stripes is computed in parallel by means of a cross-shaped window, which increases the receptive field and captures more detailed features. The edge detection auxiliary network improves the image resolution by up-sampling, which improves the quality of the edge information in the image, making the extracted edges under rich convolution finer and more complete.
- The experiments conducted on infrared image datasets demonstrate that our proposed method exhibits superior super-resolution image reconstruction performance, particularly in the edge prominent regions, compared to methods such as SwinIR, especially at an 8 × magnification factor.

## Related work

### Infrared image super-resolution

The successful application of deep learning SR models to visible light images has prompted many researchers to apply them to infrared images. However, the performance of directly applying visible light SR models to infrared images is often unsatisfactory. Inspired by the SRCNN method, Choi et al.^27^proposed the thermal enhancement network (TEN), which is trained using visible spectral data to enhance the resolution of infrared images. However, the improvement achieved is quite limited due to the differences between the two spectra. He et al.^28^proposed Cascaded deep network with multiple receptive fields for infrared image super-resolution (CDNMRF).Marivani et al.^29^proposed multimodal image SR using visible images to provide auxiliary information. Zou et al.^30^explored an infrared image super-resolution reconstruction method based on a skip connection convolutional neural network, which extracts image features through convolutional layers and recovers image details through deconvolutional layers. Prajapati et al.^26^proposed a channel splitting-based convolutional neural network (ChasNet), which utilizes channel splitting to extract high-frequency features of infrared images. Gutierrez et al.^31^designed the (AVRFN) model by combining dilated convolutions and second-order channel attention. Yang et al.^32^invented the spatial attention residual network (SAResNet), composed of spatial attention and residual blocks. Both networks aim to improve the accuracy of reconstructed images through attention mechanisms. However, Du et al.^33^abandoned the attention mechanism and achieved high reconstruction accuracy by capturing a larger receptive field through hybrid convolutions with multi-scale residuals.

### Vision transformer

Transformer was proposed by Vaswani et al.^13^ in 2017, which is a model that does not contain convolution and is entirely based on the attention mechanism. Given the successful applications of Transformer in the field of natural language processing, there has been a gradual exploration of its potential application in the field of computer vision. Dosovitskiy et al.^14^ first introduced the transformer for computer vision tasks, proposing the vision transformer (ViT). ViT performs multi-head self-attention on global feature maps, achieving promising results and demonstrating the effectiveness of transformer in the field of vision. Liu et al.^10^ proposed the Swin Transformer, which employs multi-head self-attention solely within local windows to reduce computation time. Additionally, it utilizes a shifted window mechanism to enhance information interaction between windows. Dong et al.^15^ introduced the core design of the CSWin Transformer, centred around the cross-shaped window self-attention (CSWSA) module. By parallelly executing self-attention for horizontal and vertical stripes within multiple attention heads divided into two groups, it achieves better results without increasing computational complexity.

### SwinIR

SwinIR^9^ consists of three components: a shallow feature extraction module, a deep feature extraction module, and a reconstruction module. The shallow feature extraction module employs 3 × 3 convolutional layers to extract shallow features. The deep feature extraction module is primarily composed of multiple RSTBs (residual Swin Transformer blocks) and a 3 × 3 convolutional layer for feature enhancement. Each RSTB utilizes multiple STLs (Swin Transformer layers) for local attention and interaction across different receptive fields, thereby enhancing the model's expressive capabilities. The reconstruction module integrates both shallow and deep features for image reconstruction. SwinIR integrates the features of CNN and transformer to achieve good experimental results on typical super-resolution datasets, which demonstrates the effectiveness of applying transformer to low-level visual tasks.

## Proposed method

### Network architecture

**Figure 1:**
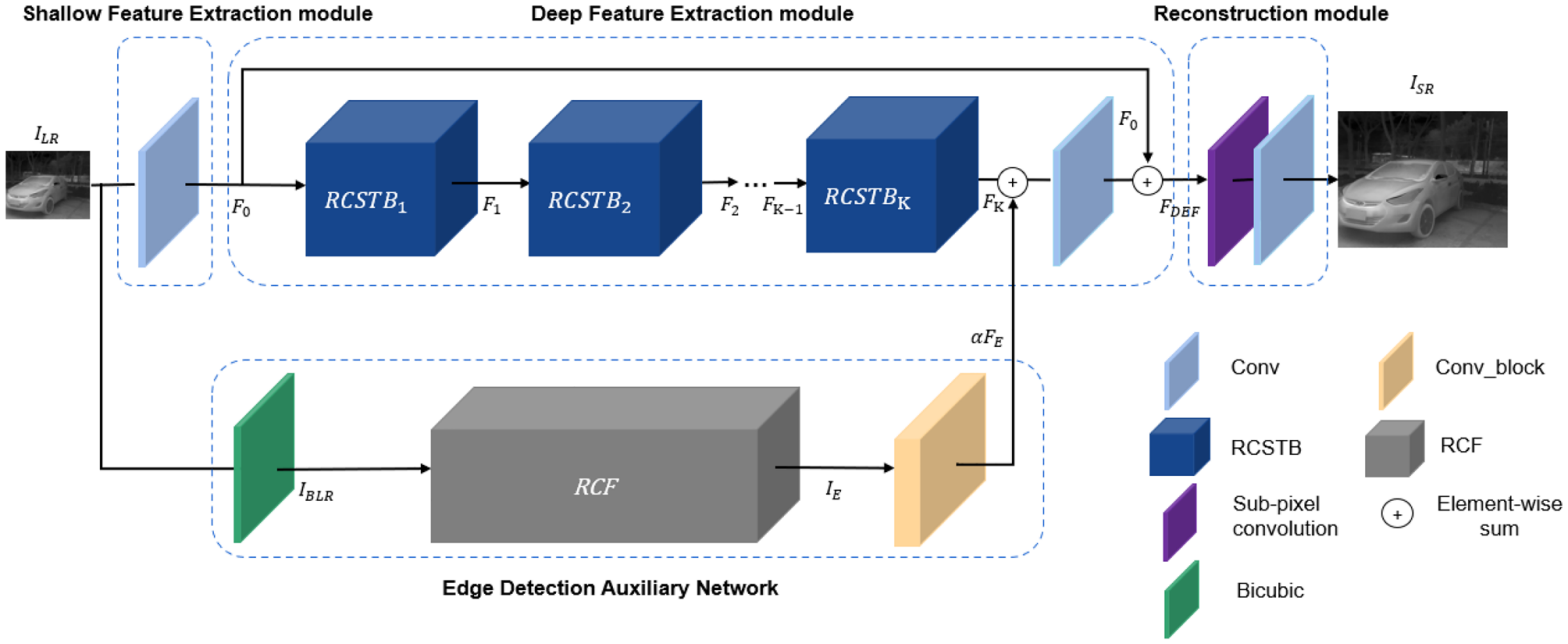
The overall structure of TESR.

The overall structure of TESR is shown in Fig. 1, and the model contains two sub-networks: the SR backbone network and the edge detection auxiliary network (EDAN), in which the SR backbone network consists of three modules: shallow feature extraction module (SFEM), deep feature extraction module (DFEM), and reconstruction module (RECM).

EDAN is composed of a bicubic upsampling layer for bilinear interpolation, a rich convolutional edge detection network RCF^16^, and a convolutional module Conv_block. EDAN performs edge extraction on the input LR image, denoted as ${I}_{\text{LR}}$, to obtain edge features ${F}_{E}$. These edge features assist the SR main network in reconstructing high-quality infrared images.

1 $${F}_{E}=B\_RCF\left({I}_{LR}\right)$$

where B_RCF(∙) denotes the EDAN.

SFEM is a convolutional layer with a convolutional kernel size of 3 × 3 used to extract the shallow feature ${F}_{0}$ from the input ${I}_{\text{LR}}$.

2 $${F}_{0}={H}_{SF}\left({I}_{LR}\right)$$

where ${H}_{SF}$ (∙) denotes the SFEM.

DFEM mainly includes high-frequency texture feature extraction and edge feature fusion. The high-frequency texture feature extraction contains K residual cross-shaped window transformer blocks (RCSTB), and the features ${F}_{1}$, ${F}_{2}$, …, ${F}_{K-1}$ and ${F}_{K}$ are extracted from the ${F}_{0}$ with the K RCSTB modules in order to obtain the high-frequency texture feature ${F}_{K}$.

3 $${F}_{i}={H}_{RCST{B}_{i}}\left({F}_{i-1}\right),i=\text{1,2},\cdot \cdot \cdot ,K$$

where ${H}_{RCST{B}_{i}}\left(\cdot \right)$ denotes the i-th RCSTB.

After combining ${F}_{K}$ and ${F}_{E}$ in DFEM, the fused features undergo refinement through a 3 × 3 convolutional layer. Subsequently, deep features ${F}_{DEF}$ are obtained by fusing the refined features with ${F}_{0}$ through long skip connections.

4 $${F}_{DEF}={H}_{CONV}\left({F}_{K}+\alpha {F}_{E}\right)+{F}_{0}$$

where ${H}_{CONV}\left(\cdot \right)$ denotes a 3 × 3 convolutional layer. The long skip connections in DFEM facilitate the fusion of low-frequency and high-frequency information, allowing DFEM to focus on the extraction of high-frequency information and edge enhancement. To enhance the edge information in ${F}_{K}$ effectively, this paper introduces a balancing factor α and overlays ${F}_{E}$ onto ${F}_{K}$ with adjusted intensity.

RECM consists of sub-pixel convolution and a 3 × 3 convolutional layer, employed to reconstruct a high-quality SR image ${I}_{SR}$ from ${F}_{DEF}$.

5 $${I}_{SR}={H}_{REC}\left({F}_{DEF}\right)$$

where ${H}_{REC}\left(\cdot \right)$ denotes the RECM.

### Residual cross-shaped window transformer block

**Figure 2:**
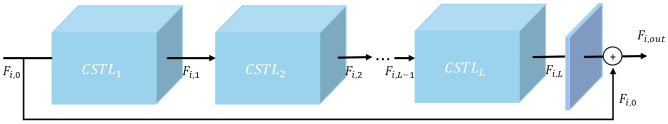
RCSTB structure map.

**Figure 3:**
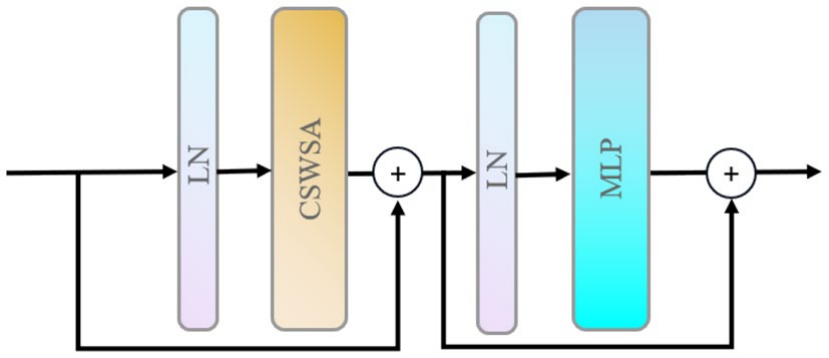
CSTL structure map.

This paper designs the RCSTB based on the CSWin Transformer, as illustrated in Fig. 2. The RCSTB is comprised of L cross-shaped window transformer layers (CSTL) and a 3 × 3 convolutional layer. The structure of CSTL is depicted in Fig. 3.

Initially, CSTLs are employed to sequentially extract features ${F}_{i,1}$, ${F}_{i,2}$, …, ${F}_{i,L}$ from the input feature ${F}_{i,0}$.

6 $${F}_{i,j}={H}_{CST{L}_{i,j}}\left({F}_{i,j-1}\right),j=\text{1,2},\cdot \cdot \cdot ,L$$

where ${H}_{CST{L}_{i,j}}\left(\cdot \right)$ denotes the j-th CSTL in the i-th RCSTB.

Subsequently, ${F}_{i,L}$ is refined using a 3 × 3 convolutional layer, and the output feature ${F}_{i,out}$ is obtained by merging it with the input feature ${F}_{i,0}$.

7 $${F}_{i,out}={H}_{CON{V}_{i}}\left({F}_{i,L}\right)+{F}_{i,0}$$

where ${H}_{CON{V}_{i}}\left(\cdot \right)$ denotes the 3 × 3 convolutional layer in the i-th RCSTB.

**Figure 4:**
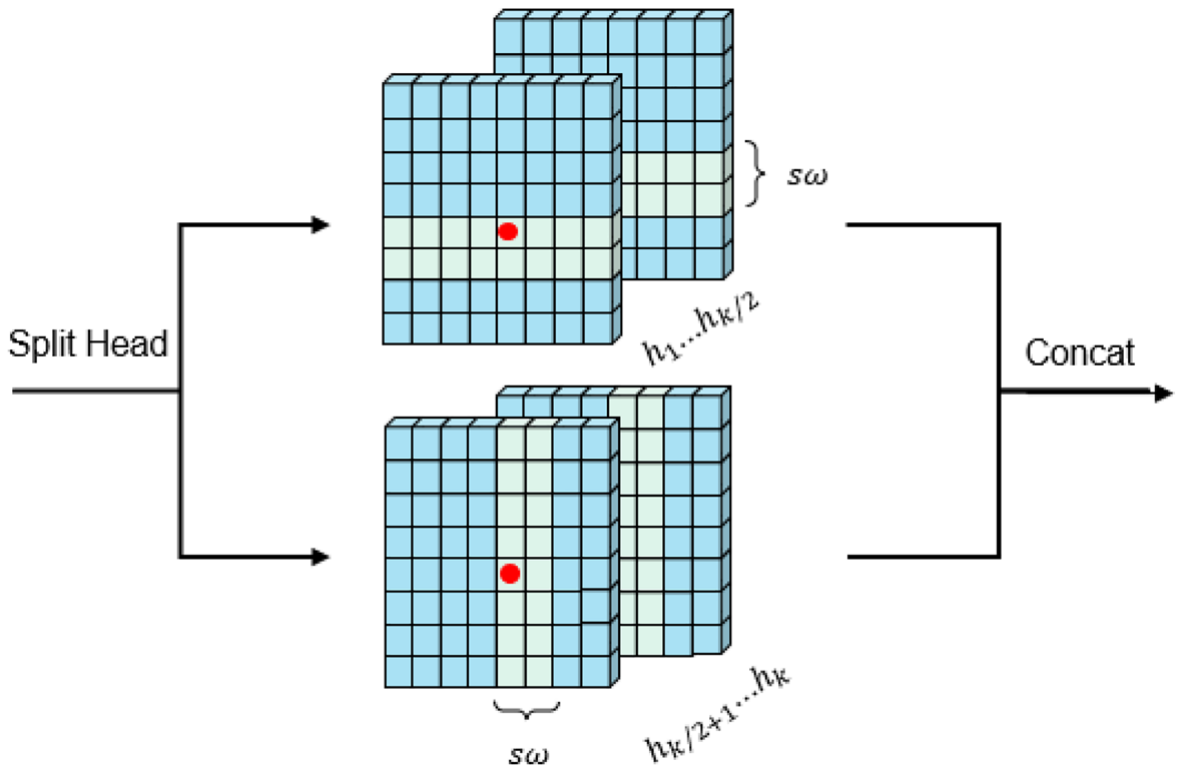
CSWSA structure map.

CSTL is based on the standard multi-head self-attention of the original transformer layer, consisting of the cross-shaped window self-attention (CSWSA) module, a multi-layer perceptron (MLP), and layer normalization (LN)^17^. There are two main improvements over the original transformer layer, CSWSA (cross-shaped window self-attention) and locally-enhanced positional encoding (LePE). CSWSA is a type of multi-head self-attention, as shown in Fig. 4, which is realised by computing horizontal and vertical stripe self-attention of width sw in parallel. Consequently, under equivalent conditions, CSWSA possesses a larger receptive field compared to traditional window-based self-attention. In addition, since important positional information usually comes from the local domain and self-attention has alignment invariance, it can lead to the frequent neglect of positional information in two-dimensional images. Hence, LePE is employed in the position encoding of CSWSA to compute and capture local positional information.

The designed RCSTB in this paper combines the adaptive filtering characteristics of Transformers with the spatially invariant filtering properties of convolution^18^ to enhance the modelling capability of TESR.

### Edge detection auxiliary network

Edge information is one of the fundamental and critical features in image processing. For an image, having well-defined edges can enhance visual impact. Therefore, preserving or introducing certain edge information can enhance the visual appeal of super-resolution (SR) images. Currently, there is a range of traditional edge feature extraction operators, such as Canny, Sobel, and so on. Additionally, there are edge extraction networks based on CNN, such as HED^19^, RCF, and others. The traditional edge detection operator Canny is sensitive to noise, easily identifying positions with significant grayscale changes caused by noise as edges. Moreover, the low-level edge information obtained is challenging to represent high-level edge details. However, RCF integrates output results from different scales, facilitating multi-scale and multi-level learning for images. This enables the model to acquire more refined high-level edge features.

**Figure 5:**
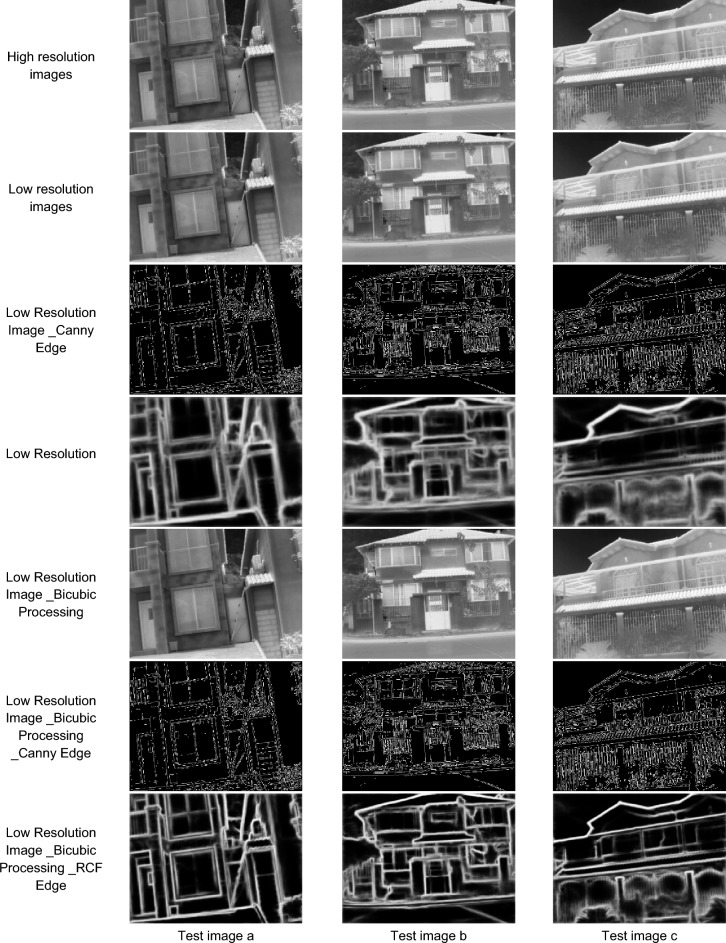
Comparison of low-resolution images (LR) and super-resolution images (LR_Bicubic processing) and the effect of edge extraction using Canny and RCF respectively.

Figure 5 illustrates a comparison of the edge extraction effects using Canny and RCF on super-resolution images and low-resolution images with a scaling factor of × 2. The edges extracted by the Canny operator (third row) contain a significant amount of noise and lack continuity. On the other hand, the edges extracted by RCF (fourth row) represent the contours of the main objects, with less noise and better continuity. Therefore, RCF is chosen as the primary method for edge extraction in this paper. When the image resolution is low, performing edge extraction on it not only results in limited information but also leads to poor continuity of edges, especially for small objects within the image. However, in the field of SR reconstruction, even basic interpolation algorithms like Bicubic can enhance the quality of an image, making the edge information more refined. As shown in Fig. 5, the edge extraction results are improved after using Bicubic interpolation, with the sixth row outperforming the third row, and the seventh row outperforming the fourth row. Therefore, Bicubic processing is applied before edge extraction in this paper.

**Figure 6:**
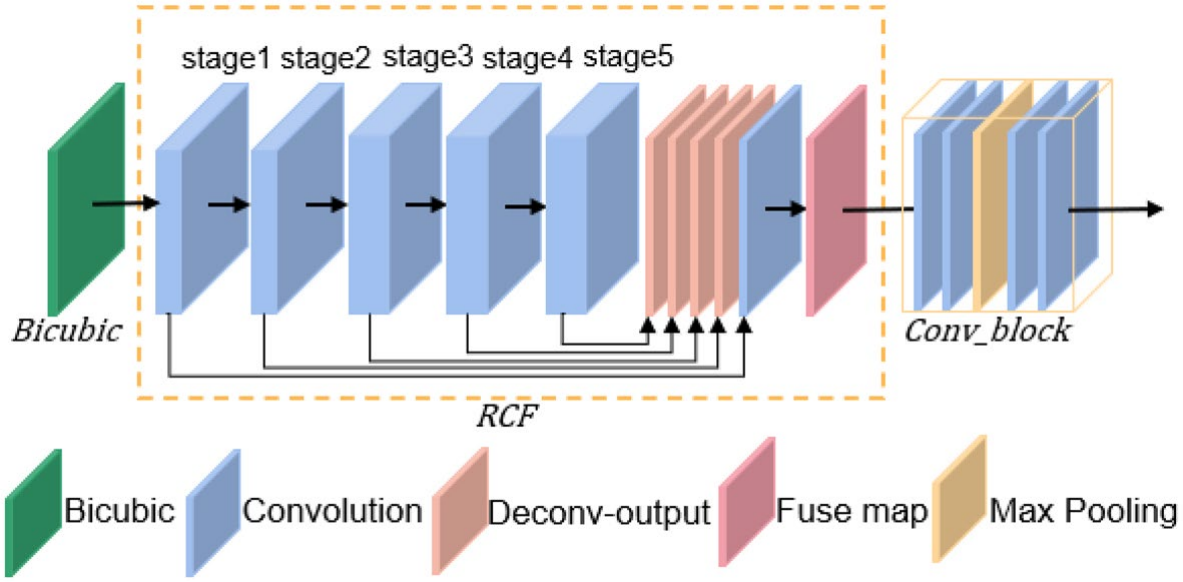
EDAN structure map.

This paper introduces an EDAN designed to assist in the reconstruction of SR images, as illustrated in Fig. 6. Firstly, the input image ${I}_{LR}$ is subjected to a 2 × Bicubic up-sampling to obtain ${I}_{BLR}$, thereby increasing the number of edge pixels in the low-resolution infrared image. Subsequently, ${I}_{BLR}$ is fed into RCF to extract edge information, resulting in the edge map ${I}_{E}$. Finally, ${I}_{E}$ undergoes refinement through Conv_block, composed of four 3 × 3 convolutional layers and one pooling layer, to obtain the edge feature ${F}_{E}$. ${F}_{E}$ will be utilized to enhance the edge information of deep features. The entire process can be expressed as follows:

8 $${I}_{BLR}=BIC\left({I}_{LR}\right)$$

9 $${I}_{E}=RCF\left({I}_{BLR}\right)$$

10 $${F}_{E}=COB\left({I}_{E}\right)$$

where $BIC\left(\cdot \right)$ denotes the Bicubic module; $RCF\left(\cdot \right)$ denotes the RCF edge detection network; $COB\left(\cdot \right)$ denotes the Conv_block.

Moreover, the low-resolution image obtained by degrading the high-resolution image may not accurately capture certain details of the original image, as exemplified by the railing portion of the LR image of test image c in Fig. 5. To avoid amplifying erroneous edge information that may affect the results of super-resolution (SR) reconstruction, this paper introduces a balancing factor $\alpha \left(0\le \alpha \le 1\right)$. Under the influence of $\alpha$, the edge information of deep features is enhanced using ${F}_{E}$, aiming to balance the conflict between edge features and deep features during the feature fusion stage. Further discussion on $\alpha$ is provided in Sect. 3.4.

## Experiments

### Datasets

In terms of experimental data, this paper utilized the datasets^20^ Thermal101 and Thermal950 for SR reconstruction of infrared images proposed by Rivadeneira et al.^21,22^ in 2019 and 2020, respectively. Due to the limited quantity of image data, the current study merged and organized these two datasets, resulting in a total of 950 training images, 50 validation images, and 50 testing images. The original image was taken as HR image data, the corresponding LR image was obtained by downsampling the HR image using Bicubic and adding the appropriate amount of Gaussian noise. To demonstrate the model's generalization, we also conducted comparative experiments with other infrared super-resolution reconstruction models using the infrared image dataset provided by Zou et al.^30^, which we simply denote as Thermal700 based on the number of images it contains.

### Training details

This study employs the PyTorch framework to construct the experimental model. The CPU used is an Intel Core i9-12900KF, with 64GB of RAM, and a single Nvidia GeForce 3090Ti GPU.

In the training phase, for EDAN, the weight parameters provided by RCF^16^ are first frozen and loaded. Subsequently, the entire TESR model is trained. Before feeding the LR images into the model for training, this paper extends the training data by rotating, translating, and flipping as a way to increase the number and diversity of training data.

Referring to the base model SwinIR, this paper sets the total number of iterations to 500,000. ${L}_{1}$ is chosen as the loss function, and Adam is employed as the optimizer with an initial learning rate of 0.0002. Learning rate decay is applied at 250,000, 400,000, 450,000, and 475,000 iterations, halving the learning rate at each decay point. The parameters RCSTB number, CSTL number, the width of horizontal or vertical stripes (sw), balance coefficient α, channel number, and the number of multi-head self-attention heads are set to 6, 6, 6, 0.1, 180, and 6, respectively. Due to the limitation of hardware devices, the batch sizes of TESR and SwinIR are set to 8 in this paper in the process of training, while keeping the other model hyperparameters consistent with their original paper.

### Evaluation metrics

In this paper, peak signal to noise ratio (PSNR) and structural similarity index (SSIM) are chosen as the error evaluation function, assessed on the Y channel after conversion to the YCbCr color space. The expressions for PSNR and SSIM are as follows:

11 $$\text{PSNR} = 10\mathit{lg}(\frac{{M}^{2}}{MSE})$$

12 $$SSIM(x,y)=\frac{(2{\mu }_{x}{\mu }_{y}+{c}_{1})(2{\mu }_{xy}+{c}_{2})}{({\mu }_{x}^{2}+{\mu }_{y}^{2}+{c}_{1})({\sigma }_{x}^{2}+{\sigma }_{y}^{2}+{c}_{2})}$$

In Eq. 11, M represents the maximum grayscale value of the image pixels, typically set to 255. MSE denotes the Mean Square Error between the reconstructed image and the high-resolution image. A higher PSNR indicates lower distortion and better quality of the generated image. In Eq. 12, ${\mu }_{x}$ and ${\mu }_{y}$ represent the means of the reconstructed image x and the high-resolution image y, respectively. ${\sigma }_{x}$ and ${\sigma }_{y}$ denote the variances of the reconstructed image x and the high-resolution image y, respectively. ${\mu }_{xy}$ is the covariance between the reconstructed image x and the high-resolution image y. ${c}_{1}$ and ${c}_{2}$ are constants. The SSIM value is directly proportional to the similarity between the reconstructed image x and the high-resolution image y, a higher SSIM value indicates greater similarity between x and y. When the two images are identical, the SSIM value is 1.

## Experimental results

To validate the effectiveness of TESR, this study selected nine representative models for comparative experiments. Among them, Bicubic represents the traditional method; SRCNN^1^, FSRCNN^23^, ESPNN^2^, VDSR^3^ , EDSR^4^, SRGAN^7^, ESRGAN^8^, RDN^24^, RCAN^6^, SwinIR^9^ and HAT^25^ are deep learning-based methods.

Tables 1, 2, and 3 present the PSNR and SSIM results on the test set under scaling factors of × 2, × 4, and × 8, respectively. In terms of quantitative metrics, our model exhibits varying degrees of improvement compared to the traditional Bicubic method and various deep learning-based methods. Specifically, the PSNR improvement ranges from 1.8017 dB to 4.1446 dB at scaling factors of × 4 and × 8, and SSIM improvement ranges from 0.0111 to 0.0447.

**Table 1:** Reconstruction results with a scale factor of × 2.

Model	Scale	PSNR	SSIM
Bicubic	× 2	42.1293	0.9816
SRGAN	× 2	42.6825	0.9831
ESRGAN	× 2	42.1034	0.9812
SRCNN	× 2	42.8671	0.9837
FSRCNN	× 2	41.5147	0.9824
ESPCNN	× 2	43.0736	0.9843
VDSR	× 2	44.2994	0.9881
EDSR	× 2	45.2888	0.9904
RDN	× 2	45.3315	0.9905
RCAN	× 2	45.9127	0.9918
SwinIR	× 2	45.8080	0.9914
HAT	× 2	**46.2683**	**0.9925**
TESR	× 2	46.2480	0.9923

Significant values are in bold.

**Table 2:** Reconstruction results with a scale factor of × 4.

Model	Scale	PSNR	SSIM
Bicubic	× 4	33.4683	0.8995
SRGAN	× 4	33.8042	0.9013
ESRGAN	× 4	33.4694	0.8997
SRCNN	× 4	33.8219	0.9039
FSRCNN	× 4	32.9525	0.9011
ESPCNN	× 4	34.0988	0.9085
VDSR	× 4	34.6714	0.9172
EDSR	× 4	35.2260	0.9263
RDN	× 4	35.2873	0.9270
RCAN	× 4	35.3393	0.9271
SwinIR	× 4	35.3988	0.9276
HAT	× 4	35.6317	0.9302
TESR	× 4	**35.6585**	**0.9307**

Significant values are in bold.

**Table 3:** Reconstruction results with a scale factor of × 8.

Model	Scale	PSNR	SSIM
Bicubic	× 8	28.3390	0.7979
SRGAN	× 8	28.6236	0.8023
ESRGAN	× 8	28.3401	0.7984
SRCNN	× 8	28.7166	0.8040
FSRCNN	× 8	26.6605	0.8021
ESPCNN	× 8	29.0909	0.8151
VDSR	× 8	29.2221	0.8198
EDSR	× 8	29.5918	0.8311
RDN	× 8	29.8739	0.8365
RCAN	× 8	29.8576	0.8376
SwinIR	× 8	29.9686	0.8378
HAT	× 8	30.1021	0.8397
TESR	× 8	**30.1407**	**0.8426**

Significant values are in bold.

Compared to other deep learning-based methods, TESR has slightly lower PSNR and SSIM metrics than HAT when the scaling factor is × 2. When the scaling factor is × 4, TESR also exhibits overall superior performance in terms of both PSNR and SSIM metrics. When the scaling factor is × 8, the TESR is even better in terms of metrics. The experimental results above indicate that TESR consistently achieves the optimal values or suboptimal values on the test dataset. Quantitative metrics have demonstrated the superiority of the proposed method.

**Figure 7:**
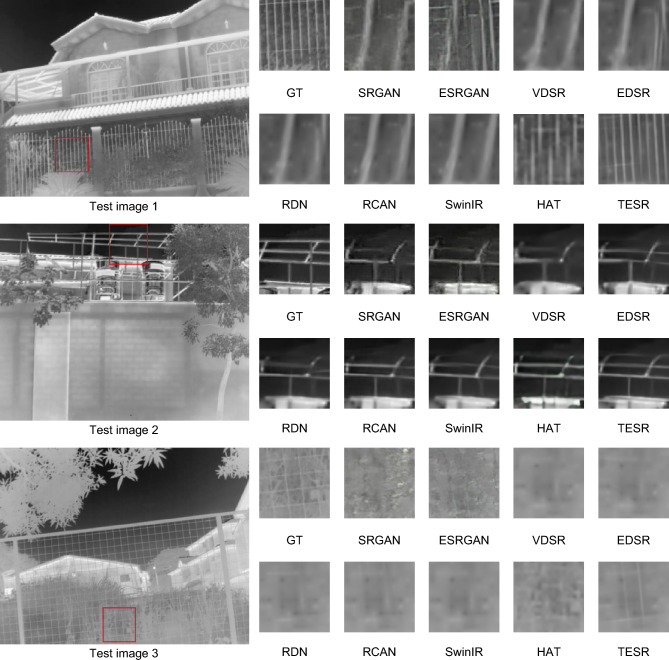
Comparison of visual effects of different methods at × 8 scale factor.

To further demonstrate the effectiveness of TESR from visual perspective. This paper selected three images from the test datasets to showcase the reconstruction results of various comparative methods at a scaling factor of × 8, as illustrated in Fig. 7. In order to provide a more intuitive display of the edge enhancement effect of our method TESR on reconstructed images, we selectively cropped portions of the reconstructed images containing long and slender objects for demonstration. The cropped sections are highlighted with red boxes. Moreover, for other scenes in the test images, the reconstruction performance of our method TESR is comparable to or even superior to other methods. Due to the significant scaling factor, it is evident from the images that the visual quality of the images reconstructed by most comparative methods is notably poor, exhibiting severe blurry artifacts. In contrast, TESR can restore more high-frequency details, achieving superior reconstruction results that closely resemble the original high-definition image (GT). For test image 1, most of the comparison methods are unable to reconstruct the railing portion, and some of them even fail to discern the conveyed information in the image. Only TESR is capable of reconstructing an image close to reality. In test image 2, earlier models such as SRGAN, ESRGAN, and VDSR produce reconstructed images that lose the main structure. On the other hand, recent methods like SwinIR and HAT can reconstruct the main contours but may not restore more detailed image edge information. Only TESR can achieve more refined results. In test image 3, other comparative methods fail to restore the clear structure of the soccer net. In contrast, the TESR model performs well in this regard.

When the scaling factor is extremely large, the information contained in the low-resolution (LR) infrared images becomes highly limited, making it challenging for super-resolution (SR) methods to reconstruct valuable results. Most comparative methods perform poorly at high magnification factors. However, the proposed TESR in this study can acquire and utilize more useful information, leading to better reconstruction results.

To further validate the effectiveness of the model on infrared images, we conducted a comprehensive comparison between several state-of-the-art infrared SR methods and the proposed TESR, including TEN^27^, CDNMRF^28^, and AVRFN^31^. The above methods were tested on three different datasets, as shown in Table 4. Across all scaling factors, TESR outperforms the other methods by a wide margin in all metrics.

**Table 4:** Quantitative comparison of various infrared SR methods on different datasets.

Model	Scale	Thermal950	Thermal101	Thermal700
		PSNR/SSIM	PSNR/SSIM	PSNR/SSIM
TEN	× 2	43.52/0.9857	40.68/0.9407	38.27/0.9520
CDNMRF	× 2	44.55/0.9888	40.83/0.9423	38.96/0.9550
AVRFN	× 2	45.05/0.9897	40.98/0.9434	39.43/0.9565
TESR	× 2	**45.94/0.9916**	**41.48/0.9487**	**40.02/0.9587**
TEN	× 4	34.28/0.9121	36.11/0.8952	29.25/0.8488
CDNMRF	× 4	34.96/0.9220	36.44/0.8997	30.18/0.8577
AVRFN	× 4	35.13/0.9239	36.49/0.9001	30.80/0.8636
TESR	× 4	**35.37/0.9270**	**36.71/0.9108**	**30.94/0.8650**
TEN	× 8	28.93/0.8129	32.26/0.8506	25.51/0.7436
CDNMRF	× 8	29.42/0.8257	32.47/0.8549	25.88/0.7569
AVRFN	× 8	29.73/0.8302	32.54/0.8544	26.44/0.7617
TESR	× 8	**29.97/0.8377**	**32.65/0.8553**	**26.57/0.7628**

Significant values are in bold.

## Ablation experiment and parameter discussion

### Ablation experiment

In order to verify the influence of incorporating edge information on the performance of infrared image super-resolution reconstruction. This paper takes SwinIR as the base model and compares the performance by introducing parameters with randomly initialized RCF and EDAN (SwinIR + NP-EDAN) and edge pre-trained RCF and EDAN (SwinIR + EDAN). The comparative results are shown in Table 5. It can be seen from Table 5 that when the magnification factor is × 4, the "SwinIR + NP-EDAN" model exhibits a performance decline compared to the baseline SwinIR model. This is attributed to the fact that the randomly initialized parameters of the RCF do not provide additional edge information during the training process and may extract some irrelevant features, thereby affecting the performance of super-resolution reconstruction. However, the "SwinIR + EDAN" model, by virtue of fixing the RCF weights, reliably provides additional edge information, thereby enhancing edge features and improving the performance of infrared image super-resolution reconstruction. Thus, it can be concluded that introducing extra edge information can enhance the effectiveness of super-resolution reconstruction.

**Table 5:** Reconstruction performance comparison of SwinIR introducing pre-trained RCF and non-pre-trained RCF.

Scale	Model	PSNR	SSIM
× 4	SwinIR	35.3988	0.9276
	SwinIR + NP-EDAN	35.3896	0.9272
	SwinIR + EDAN	**35.4859**	**0.9288**

Significant values are in bold.

To substantiate the positive impact of CSTL and EDAN on TESR, this paper conducts ablation experiments on CSTL and EDAN with SwinIR as the base model while keeping the model parameters consistent. The results are shown in Table 6.

**Table 6:** Results of ablation experiments.

Scale	Model	CSTL	EDAN	PSNR	SSIM
× 2	SwinIR			45.8080	0.9914
	TESR	√		45.8716	0.9915
	TESR		√	46.2376	0.9922
	TESR	√	√	**46.2480**	**0.9923**
× 4	SwinIR			35.3988	0.9276
	TESR	√		35.5687	0.9299
	TESR		√	35.4859	0.9288
	TESR	√	√	**35.6585**	**0.9307**
× 8	SwinIR			29.9686	0.8378
	TESR	√		30.1219	0.8420
	TESR		√	29.9897	0.8385
	TESR	√	√	**30.1407**	**0.8426**

Significant values are in bold.

**Figure 8:**
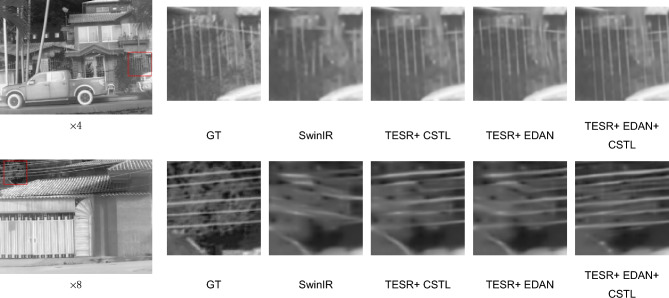
Visual comparison of ablation experiments at scaling factors of × 4 and × 8.

From Table 6, it can be observed that at scaling factors × 2, × 4, and × 8, SwinIR achieves PSNR values of 45.8080dB, 35.3988dB, and 29.9686dB, respectively, with corresponding SSIM values of 0.9914, 0.9276, and 0.8378. When CSTL is used alone, compared to the base model, the PSNR improves by 0.0636dB, 0.1699dB, and 0.1533dB at the three scaling factors, while the SSIM improves by 0.0001, 0.0023, and 0.0042, respectively. This is because CSTL has a larger receptive field, allowing the model to leverage deeper-level features and consequently achieve better reconstruction results. When EDAN is added independently at the three scaling factors, although it introduces some additional parameters, the performance improvement is noticeable. The PSNR increases by 0.4296dB, 0.0871dB, and 0.0211dB, while the SSIM increases by 0.0008, 0.0012, and 0.0007, respectively. The primary reason is that EDAN can enhance image edge information to help image reconstruction. When CSTL and EDAN are applied simultaneously, there is a further improvement in objective metrics. Because it is less difficult to reconstruct at a scaling factor of × 2, there is no significant difference between the ablation results. We list the results for scaling factors of × 4 and × 8 in Fig. 8 . It can be clearly seen that using CSTL and EDAN simultaneously can reconstruct clear contours without distortion.

### Parameter discussion

In CSWSA, the width (sw) of horizontal or vertical stripes is closely related to the size of the receptive field. Table 7 demonstrates the impact of sw on model performance at a magnification factor of × 4, revealing a positive correlation between PSNR, SSIM, and sw. This is because a wider stripe width can increase the model's receptive field, alleviating the issue of information loss due to the depth of the network. To balance learning capacity and computational complexity, sw is set to 6 in this paper.

**Table 7:** Results of $sw$ size discussion.

Scale	$sw$	PSNR	SSIM
× 4	2	35.4721	0.9286
	4	35.5906	0.9302
	6	**35.6585**	**0.9307**

Significant values are in bold.

In the process of SR reconstruction, the intensity of edge features can impact the reconstruction results, necessitating a discussion on the parameter α. In this paper, α is sequentially set to 0, 0.01, 0.1, and 1. Table 8 demonstrates the influence of α on model performance at a magnification factor of × 4. From the table, it is evident that when α is set to 0.1, both PSNR and SSIM reach their maximum values. Therefore, α is set to 0.1 in this paper.

**Table 8:** The results of the discussion on the value of $\alpha$.

Scale	$\alpha$	PSNR	SSIM
× 4	0	35.6385	0.9303
	0.01	35.6124	0.9303
	0.1	**35.6585**	**0.9307**
	1	35.6142	0.9304

Significant values are in bold.

Considering the issue of the total model parameter count, although stacking RCSTB blocks can enhance the network's modeling capability, the improvement diminishes once a certain threshold is reached. Therefore, we discuss the impact of the parameter k on the parameter count and model performance. Table 9 shows the impact of the parameter k on model performance at a scaling factor of × 4. From the table, it can be observed that performance improvements are marginal beyond k = 4, while the number of parameters is still stacked normally. Therefore, k is set to 4 in this paper.

**Table 9:** The results of the discussion on the value of k.

Scale	$k$	1	2	3	4	5	6
× 4	Parameters(M)	6.8	7.1	7.5	**8.1**	8.9	9.7
	PSNR	35.1317	35.381	35.5902	**35.6585**	35.6613	35.6624
	SSIM	0.9256	0.9271	0.9298	**0.9307**	0.9307	0.9308

Significant values are in bold.

## Conclusion

In this paper, we introduce a transformer-based edge-enhanced super-resolution model (TESR) for infrared image super-resolution reconstruction tasks. The CSWin Transformer layer in this model exhibits excellent long-range contextual modelling and global information capture capabilities. It can parallelly compute self-attention for horizontal and vertical stripe patterns, achieving improved reconstruction results without increasing computational complexity. Additionally, the proposed edge detection auxiliary network can extract fine-grained edge information. Using this edge information as supplementary data enhances the edges of the reconstructed infrared images. The experimental results indicate that our model outperforms current representative methods in terms of objective evaluation metrics, including PSNR and SSIM. In terms of subjective visual effects, our model demonstrates the ability to recover more high-frequency details, resulting in images with clearer edges. It is worth noting that because of the use of transformer as the main architecture and the introduction of an edge detection auxiliary network, the computational and parameter complexity of the network is relatively high. Future work will focus on optimizing the model to address this issue, enabling a lightweight version that maintains superior performance.

## Data Availability

The datasets used and analysed during the current study available from the corresponding author on reasonable request.
